# Supplementary material for: Big Data and Machine Learning to Improve European Grapevine Moth (Lobesia botrana) Predictions
Source: Plants (Basel). 2023 Feb 1;12(3):633. doi: 10.3390/plants12030633 (PMC9921845; doi:10.3390/plants12030633)
Supplement: Supplementary file 1 [file plants-12-00633-s001.zip › plants-2147490-supplementary.pdf]

Big Data and Machine Learning to Improve European Grapevine Moth (*Lobesia botrana*) Predictions

J. Balduque-Gil, F.J. Lacueva-Pérez, G. Labata-Lezaun, R. del-Hoyo-Alonso, S. Ilarri, E. Sánchez-Hernández, P. Martín-Ramos, and J.J. Barriuso-Vargas

SUPPORTING INFORMATION

Table S1. Parameters recorded daily every 30 minutes by the weather stations used in the modeling.

Parameter	Measurement unit
Temperature	°C
Relative humidity	%
Wind speed	m/s
Wind direction	°
Solar radiation	W/m^2
Accumulated precipitation	mm

Table S2. List of SiAR weather stations used for modeling.

Code	Longitude	Latitude	Altitude
Z01	−1.329722	41.451944	395
Z05	−1.281944	41.583056	327
Z14	−1.507500	41.855000	376
Z15	−1.745833	41.916111	521
Z18	−1.424444	41.108056	747
Z22	−1.249444	41.843056	226
Z24	−1.614620	41.362900	523

Table S3. Stages of development and sum of cumulative temperatures required according to the Touzeau model.

Stage	Temperature Sum (*Tou*)	Accumulated Temperature Sum (*Tou*)
Maximum flight 1st Generation	125	125
Egg eclosion 1st Generation	75	200
Larval development	170	370
Pupal formation and adult appearance of 2nd Generation	130	500
Egg eclosion 2nd Generation	65	565
Larval development	255	820
Chrysalis formation and adult appearance of 3rd Generation	130	950
Egg eclosion 3rd Generation	75	1025

Table S4. Monitoring sites used for validation of the Touzeau model.

Code	Altitude (m)	Latitude	Longitude
50004A03400175	671	41.34436	−1.28833
50024A00300013	435	41.43628	−1.30584
50073A00700043	530	41.38521	−1.19748
50073A01000009	632	41.35815	−1.16129
50073A02700014	626	41.33168	−1.24124
50073A03400128	556	41.37100	−1.24282
50073A03500094	493	41.40490	−1.23058
50073A04100021	517	41.39011	−1.20791
50073A04800016	582	41.36016	−1.18331
50073A05800042	664	41.32108	−1.17023
50073A08400031	565	41.35638	−1.20710
50073A08900011	616	41.33819	−1.18215
50073A09500073	613	41.32603	−1.20547
50073A10000051	595	41.33764	−1.21614
50098A03000027	719	41.29084	−1.25558
50201A00200124	702	41.29448	−1.22502
50201A02200012	716	41.28343	−1.20595
50268A00100050	657	41.34200	−1.13889

Table S5. Monitoring sites used for validation of the ML models.

Code	Altitude (m)	Latitude	Longitude
50002A00400047	860	41.17790	−1.60215
50006A03800131	571	41.76361	−1.54832
50046A01700135	669	41.31718	−1.52381
50055A02700794	400	41.85982	−1.52577
50055A05900072	515	41.81587	−1.58083
50061A00700009	434	41.80252	−1.49993
50073A01000013	613	41.35465	−1.16592
50073A04800016	582	41.36016	−1.18331
50073A07600105	556	41.35884	−1.23050
50088A00400051	528	41.39414	−1.27336
50088A02900241	578	41.37768	−1.26875
50144A50100067	588	41.38704	−1.13643
50252A03500186	730	41.68100	−1.52724
50252A03500186	730	41.68100	−1.52724
50252A03500186	730	41.68100	−1.52724
50284A01000052	635	41.25696	−1.60230
50284A01000052	635	41.25696	−1.60230
50295A03000033	638	41.36349	−1.08042
